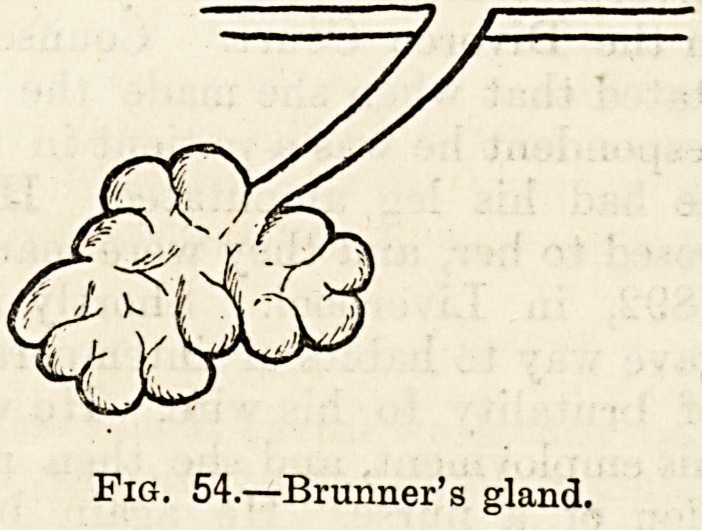# The Hospital. Nursing Section

**Published:** 1902-08-09

**Authors:** 


### The Hospital.

### Nursing Section.

Contributions for this Section of "The Hospital" should be addressed to the Editor, "The Hospital" Nursing Section, 28 & 29 Southampton Street, Strand, London, W.C.

No. 828.—Vol. XXXII. *SATURDAY, AUGUST* 9, 1902.

### THE PRESENTATION OF MEDALS BY THE QUEEN.

The ceremony of the presentation by Queen Alexandra of medals to the staff of the Imperial Yeomanry Hospitals on Monday afternoon in the gardens of Devonshire House will, it is understood, be exceedingly simple, and there will be no speeches. About 200 doctors, dressers, nurses, and orderlies will receive the distinction from Her Majesty, who, it will be recollected, accepted the post of President of the hospitals when she was Princess of Wales, and retained the post upon the accession of the King. It will be very satisfactory to the nurses who have been attached to the organisation to learn that, after payment of all expenses, Lady Howe has in hand a sufficient balance to enable the committee to form plans for starting a training home for the children of Imperial Yeomen incapacitated by the war, in which they will be taught useful arts and industries.

### NURSING QUESTIONS IN PARLIAMENT.

Last week, in the House of Commons, Mr. C. Douglas asked the Secretary of State for War whether any reward or distinction, other than the South African War medal, had been given to any of the nurses who served in South Africa; if so, whether such reward had been conferred upon Army nurses only or upon volunteers also, and, if any difference had been made in the treatment of those classes of nurses, what were the grounds upon which this had been done. Mr. Brodrick, in reply, said that the Royal Red Cross had been awarded to certain Army and Volunteer nurses who had been officially recognised. Distinctions of various grades had also, he added, been awarded by the Grand Prior of the Order of St. John of Jerusalem on the recommendation of the War Office. In the debate in Committee on the Irish Local Government No. 2 Bill, Mr. Hayden moved an amendment to provide that half the salary of any midwife or trained nurse employed under the Irish Medical Acts shall be paid out of the Local Taxation Account. Mr. Dillon, who supported the amendment, said he was glad that the Local Government Board had issued a sealed order compelling the boards of guardians to appoint trained midwives to attend outdoor cases, but owing to the small salary the guardians were able to offer, the positions were generally unfilled. Mr. Wyndham, the Irish Secretary, while expressing his hope that the clause would be allowed to go through in its present form, promised that in the recess he would consider, with the assistance of members from Ireland, the whole question of the charges which ought to be properly placed on the Local Taxation Account. This undertaking was accepted as satisfactory, and the amendment was, by leave, withdrawn.

### NURSES FROM SOUTH AFRICA.

The members of Queen Alexandra's Imperial Military Nursing Service, who returned from South Africa last week, were on the *Victoria*—Sister A. R. Chitty, who is going to resign, and Sister S. Lees, who was locally engaged for the voyage; on the *Sicilia* Sisters N. C. Crosbey and A. F. Hobbs on duty for voyage home, but do not return to South Africa; on the *Avoca*, Superintendent Sister J. Stefenson, Sisters A. G. Rees, M. E. Buse, J. K. Szczepauska, L. E. Snape, S. Horsley, J. J. Walworth, A. F. Byers, and E. Nixon, time expired, and Sisters Maclean, Parker, MacIntyre, and Mavins, invalids; on the *Carisbrooke Castle*, Sisters J. B. B. Bell and A. B. Wohlmann, neither of whom return to South Africa, owing to reduction of establishment; and on the *Columbian*, Sisters M. C. Fraser and F. M. Donald, time expired. This week the *Staffordshire*, with Sisters M. Limey and A. A. Knight on board; and the *Malta*, with Sisters A. Guthrie and A. J. Moffatt, arrived. Neither of these nurses will return to South Africa. The *Kildonan Castle*, which is due at Southampton on Saturday, has on board Sisters E. Heaton-Cole, E. Andrews, A. Ainsworth, B. E. Hutchinson, J. B. Boots, M. A. McDonald, and D. E. Reynolds; and the *Tintagel Castle*, which is due next week, Sisters A. R. Andre, A. J. Davidson, F. Greystone, and A. Von Dix.

### REDUCTION OF THE AGE LIMIT.

We are officially informed that the committee of the Royal Devon and Exeter Hospital have decided to reduce the age limit for probationers entering that institution from 23 to 21. The result of this experiment will be watched with interest. It is not, perhaps, rash to assume that the change is due to a difficulty in obtaining a sufficient number of applicants for vacancies. None the less, it is evident that if such a course should be generally adopted it would make a great difference in the supply of nurses to many a hospital.

### THE HOUSING OF NURSES.

In our correspondence columns, "A London Nurse" makes a complaint which, so far as the first part of the letter is concerned, we print only in order to show the ignorance of people who might fairly be supposed to know what is going on in the nursing world. It is inconceivable that "A London Nurse," who avers that, except in "a very few instances" no efforts have been made to improve the housing of nurses, can have read the accounts which have appeared from time to time in our columns, not only of the splendid homes for nurses opened in connection with hospitals and infirmaries, both in London and in other great cities, but also of the comfortable establishments in many of the small towns. It is true that she proceeds to dwell on the alleged inadequate accommodation provided for nurses in West End surgical homes. On this point she may be right, though if a nurse stays in one of these establishments in spite of the fact that she is not properly treated, she has only herself to blame. Her letter tends to throw fresh light on the kind of information which is considered sufficient to warrant attacks, in monthly periodicals, upon hospital managers and matrons.

### THE HOSPITAL MATRON'S QUARTERS.

We understand that the new matron of University College Hospital, in succession to Miss Hamilton, is to have her office and private apartments in the Nurses' Home. To this arrangement some exception is taken at Gower Street, and in ordinary circumstances, there is no doubt that it is advisable for the matron to have her quarters in the hospital. But as a rule the Home is some considerable distance from the main building; in the case of University College, it is for all practical purposes in the hospital, though, of course, quite separated from it, for the Home is as near to the wards as the wards are to each other; and we think that the arrangement for the matron to reside in the Home with the staff is both suitable and convenient.

### MALE NURSES AND CO-OPERATION.

The illuminating report of a squabble between rival male nurses' co-operations which we give to-day, will be perused with amused interest by our readers. It is only fair to notice that so far as the nurses connected with both the organisations are concerned, there was no suggestion of incompetence, although two attendants from an asylum in Scotland seem to have been engaged with very slender references as to character. The very often erroneous idea that men have better business heads than women is not borne out by the disclosure that although Mr. Walshe's nurses joined a "Co-operation," they did not ascertain how the co-operative principle was applied. This is a matter of vital importance, especially in view of the profits of a proprietor who, from the amount of the weekly sums paid to him by the nurses, was calculated by the counsel in his opening statement to be £3,228 a year.

### THE NURSES OF BRENTFORD UNION INFIRMARY.

The report of the sixth year of the Training School at Isleworth Infirmary, shows that since July 1st, 1901, six staff nurses who had completed their training have left to take up various appointments, and that seven probationers have been promoted to be staff nurses. There are now in the Home 23 staff nurses and probationers. One is in her fifth year, two are in their fourth, eight in their third, five in their second and seven in their first year. Lectures and classes have been held by the medical superintendent, the assistant medical officer, the matron, and the assistant matron. These were somewhat interrupted in the spring by the epidemic of influenza, so that the examinations have taken place somewhat later this year than usual. Dr. Fooks and Dr. Silvester held the annual examination for all the nurses and probationers at the end of the course of lectures July 9th and 10th. Dr. Seymour Sharkey, of St. Thomas's Hospital, conducted the fourth final examination for the nurses in their third year, July 24th. He reports that the nurses "acquitted themselves very well indeed, and showed that they had been excellently taught." Ten nurses who received instruction in massage have been successful in passing the examination and obtaining the certificate of the Incorporated Society of Trained Masseuses during the year.

### THE LATE MATRON OF BELPER ISOLATION HOSPITAL.

It is a pity that the committee having the control of Belper Joint Isolation Hospital did not agree unanimously to the testimonial which has at last been granted to Miss Martin, the late matron. The majority, we are glad to observe, included Dr. Hooper, who drew up the testimonial to the following effect:—

> "The Hospital Committee are pleased to testify to the great skill, kindness and competence shown by Miss Martin during the fourteen months she acted as Matron under them; there can be no doubt that the great success of the Isolation Hospital (as seen in its low death rate) as a means of cure for infectious diseases, and of preventing their spreading, was largely due to her untiring energy on behalf of the patients, faithfully carrying out instructions ordered, to her kind thoughtfulness for their individual comfort, and to her excellent management of the Hospital and of all who were serving under her; it may be truly said of her that she was proud of the hospital, and did her best to make it a success."

With this unequivocal acknowledgement of her services Miss Martin may well be content, but no satisfactory answer was given to the pertinent inquiry of Mr. Booth, who wanted to know "why Nurse Martin had been kept out of her testimonial for six months?" So far back as April 2nd Miss Martin's solicitors wrote to the committee pointing out that "the delay in letting their client have the promised testimonial was of serious consequence to her, as it prevented her from applying for and obtaining a position as matron in any hospital where there was a vacancy." The procrastination of the committee in the matter seems all the more reprehensible in the light of the terms of the testimonial to which the majority assented.

### BAZAAR AT CREDITON.

For the past 16 years most useful work has been done by the Nursing Association at Crediton among the sick poor of the town. Lady Audrey Buller has been President of the Association ever since its formation, and has shown great interest in its progress. At her suggestion last week, a grand bazaar was held in the beautiful grounds, lent by Mr. B. H. Hill, of Newcombes, the purpose of the effort being to secure additional funds for the maintenance of the second nurse established last year for maternity work. The opening ceremony was performed by Lady Beatrice Pole-Carew, who was introduced by General Sir Redvers Buller, V.C. Acknowledging a vote of thanks to her, General Sir Reginald Pole-Carew said that she felt amply repaid if she could be the means of doing the slightest good to so excellent an institution. The weather was somewhat unfavourable, but the attendance was large, and the stallholders did good business. The members of the Association were responsible for providing one of the six stalls. There were also amateur theatricals and many other attractions. The day's receipts exceeded £160, so that the Association's funds will substantially benefit, and the committee will, no doubt, feel justified in making the appointment of the midwifery nurse permanent, as her usefulness in the district has been readily recognised.

### HEREFORDSHIRE GENERAL HOSPITAL.

At the quarterly meeting of the governors of Herefordshire General Hospital two distinct questions of importance were raised. One was that of the extension of the private nursing staff. The matron reports that, for want of sufficient nurses, she was frequently unable to comply with applications for their services, with the result that medical men in the county complain that they have to obtain nurses from Gloucester, Worcester, Bristol, Birmingham, and other places. This, it was urged by one of the speakers at the meeting, deprives Hereford of benefits arising therefrom, direct and indirect, the latter in the form of possible legacies and gifts from grateful patients. As it also appears that the private nursing account shows an increasing profit of upwards of £300 a year, it would surely be good policy to augment the staff. It is also alleged that the accommodation provided for the nurses and servants at the hospital is inadequate. One of the governors contended that the consideration of the drainage question "was the only pressing point." If there are defects of drainage, they should, of course, be remedied immediately, but it is of equal moment that the nurses should be comfortably housed by day and night, and we are pleased to see that other governors recognise the importance of the point.

### A NURSE'S UNHAPPY MARRIAGE.

Last week Mr. Justice Barnes granted a decree nisi to Mrs. Alice Burke, formerly a nurse in the Liverpool Royal Infirmary. A sad story was told in the Divorce Court. Counsel for the petitioner stated that when she made the acquaintance of the respondent he was a patient in the infirmary, where he had his leg amputated. He subsequently proposed to her, and they were married on January 1st, 1892, in Liverpool. Shortly afterwards the man gave way to habits of intemperance, and was guilty of brutality to his wife. He was discharged from his employment, and she then resumed the occupation of a nurse. He again behaved with cruelty towards her, and on one occasion cut her head open with a piece of coal. Subsequently, he was convicted at Liverpool of obtaining money under false pretences, and, proceeding to London, went to live with another woman. Mrs. Burke, in the course of her evidence, said that the assaults upon her by her husband were of "daily occurrence."

### SWANSEA NURSING ASSOCIATION.

The new home of the Swansea District Nursing Association, which is affiliated to Queen Victoria's Jubilee Institute for Nurses, has been opened by Lady Llewelyn, who, in her speech on the occasion, said that it was entirely for the poor, and she hoped that the people of the town would put their hands in their pockets to support it. The Swansea Board of Guardians have been asked to increase their subscription, but as they already contribute £75 a year towards the support of the Nursing Association, this seems a little unreasonable. If, as Dr. Nelson Jones avers, the home, which starts with a staff of three nurses, is to prove "one of the greatest boons and blessings the people of Swansea ever had," there should be no difficulty in obtaining from individual residents the further amount required.

### A START AT UTTOXETER.

At a meeting of the residents of Uttoxeter in the Town Hall, last month, it was decided to engage a nurse to act under the direction of the representative committee which has been formed. The ladies of the committee have for some little time been actively engaged in collecting contributions, and there is now in hand, or promised, the sum of £60. This is sufficient to justify a start. We observe, however, that it is proposed to obtain a portion of the requisite income from the people who need the services of a nurse. The better plan would be not to ask for fees from the sick poor, but to invite them to subscribe a small sum regularly to the maintenance of the nursing organisation.

### PITLOCHRY NURSING HOME.

The nursing home created in connection with the Pitlochry District Nursing Association was opened last week. The building, which is a memorial to the late Dr. W. Stewart Irvine, whose popularity on the whole countryside was phenomenal, consists of male and female wards, with separate bath-rooms and other conveniences, nurses' rooms, kitchens and offices. The ceilings are lofty, and the internal arrangements have been carried out on modern principles. The cost of the home and the furnishing is £1,376, which leaves a balance in hand of the amount subscribed. A novel feature in connection with the opening ceremony, was that every visitor was invited to bring one or more pounds of groceries as a contribution towards the housekeeping of the home, with the result that quite a large stock of goods was secured. There was also at the close of the proceedings an interesting private function, namely, the presentation to Miss Anderson of a handsome writing bureau, in recognition of her valued services as district nurse during the past six years.

### THE PLUCK OF AN INFIRMARY NURSE.

On Tuesday a youth named Joseph Padain, of Poole Street, Hackney, was charged at the North London Police Court with stealing a gold watch and chain from Miss Florence Shepperd, a nurse at Bethnal Green Infirmary. The prosecutrix stated that on Monday evening, in company with another nurse, Miss Florence Bradley, she was walking down Mare Street, Hackney, when Miss Bradley felt a hand touch her, and directly afterwards saw Padain run across the road with a watch and chain in his hand; she gave chase and captured him, and though he struggled with her, she kept hold of him until a tramway-car conductor came to her assistance. The watch and chain were found to belong to Miss Shepperd. The first plea of the prisoner, who was remanded for inquiries, was that the watch was hanging out of the lady's pocket, but in court he said that it fell out of her pocket, and he was going to give it to her. In any case, Miss Shepperd is to be congratulated upon her pluck and presence of mind in the matter.

### Lectures to Nurses on Anatomy.

By W. JOHNSON SMITH, F.R.C.S., Principal Medical Officer, Seamen's Hospital, Greenwich.

### LECTURE XXIII.—THE ORGANS OF DIGESTION.

ON the right side of the pylorus is the commencement of the intestinal canal which is divided into the *small* and the *large* intestines (fig. 51). The convoluted and mobile mass of the small intestines (*i*) which is about 26 feet in length, from four to five times the length of the body, is subdivided into duodenum, a curved and fixed portion of intestine about 10 inches in length, the jejunum measuring about 10 feet, and the ileum about 15 feet.

The large intestine which commences in the right flank is much more fixed than the small intestine. Like the small intestine, it is divided into three portions: the cæcum to which is attached the thin worm-like process called the *appendix* (fig. 51, *va*), the rudimentary organ which, when inflamed, gives rise to the serious troubles classed under the title of appendicitis; the colon which ascends along the right side of the abdomen to the liver—*the ascending colon* (*a c*), then passes across to the left side—*transverse colon* (*t c*), and finally descends on this side from the spleen to the left side of the pelvis—*the descending colon* (*d c*), which near its termination is much convoluted, forming the *sigmoid flexure* (*i c*). The terminal portion of the large intestine, and of the whole alimentary canal is the *rectum* (*r*), measuring from 6 to 8 inches in length.

The wall of the digestive canal from the lower end of the gullet to the outer opening of the rectum varies much in thickness at different parts, being thickest in the stomach and large intestine, and thinnest in the lower part of the small intestine.

This wall is made up of different layers: (*a*) an external or *serous* layer of *peritoneum*; (*b*) an internal layer or *mucous* layer of mucous membrane; and (*c*) an intermediate or *muscular* layer of smooth and involuntary muscular tissue. Between the middle and the internal layer is interposed a thin sheet of connective tissue in which the blood vessels of the intestine effect their ultimate sub-division before supplying the mucous membrane and the numerous important though minute glands scattered in some parts of this layer.

The *serous* coat, consisting of peritoneum, thin and transparent like the pleural coat of the lungs, is, as has already been stated, continuous with the inner lining of the walls of the abdomen, and with the numerous bands termed ligaments and mesenteries that support and suspend the many abdominal organs. This reflected serous coat completely surrounds the stomach and also the intestines, except at a portion of the duodenum, and at the lower portion of the rectum, also, though not invariably, at portions of the ascending and descending colon.

The muscular coating is arrranged throughout the abdominal portion of the alimentary tube in two layers; an external one in which the fibres are arranged *longitudinally* or in the long *axis* of the tube, and an internal one in which the fibres take a *circular* direction. In the stomach there is a third layer, consisting of oblique fibres, and at the pyloric orifice between the cavity of the stomach and the beginning of the duodenum the circular muscular fibres are concentrated so as to form a thick and strong muscular ring. In the small intestine the longitudinal fibres form a continuous layer around the whole circumference of the bowel, and consequently the surface of this portion of the canal is smooth and even. In the cœcum and colon, however, the longitudinal fibres are concentrated into three distinct bands, extending along the whole length of these portions of the large intestine. As these thick and strong bands of muscle draw up the bowel, or prevent it from being stretched to its full length, the coating of the cœcum and colon present an alternating series of large dilatation and narrow constrictions, and this portion of intestine is, to use the proper anatomical term, sacculated.

The whole of the alimentary tract is lined by a soft, red and vascular membrane, called mucous membrane, continuous with the lining of the mouth which presents a good example of this structure.

The surface of the mucous membrance of the stomach is smooth and even when this organ is full and distended, and thrown into longitudinal folds when it is empty and contracted. It is studded in every part by the very minute openings of secreting tubules which discharge into the cavity of the stomach thick mucus and gastric fluid containing hydrochloric acid and pepsin, by the action of which the fleshy or albuminous elements of the food are digested.

The vascular mucous membrane of the small intestines from the upper part of the duodenum to about the middle of the ileum is projected in well-marked transverse folds running over from one-half to two-thirds of the circle of the intestine. These folds, termed *valvulæ conniventes*, increase to a considerable extent the surface of mucous membrane over which the liquid and digested food passes in its course from the stomach to the beginning of the large intestine. This extent is still further increased by the presence of innumerable minute processes called *villi*, resembling the "pile" of velvet. These villi serve to absorb from the alimentary canal the fatty portions of the food. The mucous membrane, like that of the stomach, is richly supplied with glandular structures, some, the simple *crypts of Lieberkuhn* (fig. 53),

FIG. 53.—Lieberkuhn's follicle or crypt.

FIG. 54.—Brunner's gland.

being found in every part of the small intestines, some, the small *racemose* or bunched glands of Brunner (fig. 54), existing only in small quantity and confined to the duodenum and others, existing in the form of minute blind sacs, either scattered as isolated bodies over every part of the small intestine, or collected together to form large and conspicuous patches—Peyer's patches or glands—which, when inflamed and ulcerated in an attack of enteric fever, cause some of the most grave and troublesome symptoms of this affection.

The coat of the duodenum at a point about 3½ inches below the pyloric end of the stomach is perforated by a wide duct which discharges into the intestinal canal the secretions of two large glands—the liver and the pancreas.

The food dissolved and chemically altered by the action of the different fluids formed by the salivary glands, by the small glands of the stomach and small intestine, and by the liver and pancreas, and converted in the first place into

### LECTURES TO NURSES ON ANATOMY.—*Continued.*

pulp called *chyme*, and afterwards into a turbid fluid called *chyle*, is removed from the alimentary tube by special vessels called lacteals and also by the intestinal veins, and finally becomes blood.

There is one important point to be remembered in connection with the anatomy of the organs of digestion. The blood collected *by the veins* from the long tract of intestine—about from nine to ten yards in extent—is not carried directly by enlarging veins to the right side of the heart, like venous blood from other organs, but before reaching the vena cava is collected in one large vessel called the *portal vein* which enters the liver and there divides and sub-divides into branches and finally into capillaries. The blood is collected from the capillaries and passed on to the vena cava and the heart by the *hepatic veins*. Thus we find in the organism a minor and subordinate circulatory scheme which is termed the *portal circulation*.

The mucous membrane of the large intestine is smooth and does not present the velvety appearance of the inner surface of the small intestine. It is studded throughout by the orifices of minute tubules or *follicles*, and, in the commencing portion—the cœcum—by small saccular glands. There are no distinct folds of mucous membrane analagous to the valvulæconniventes of the small intestine, but projections may be seen involving all the *coats* formed by the constrictions between the dilated portions or sacculi of the bowel.

### A Bank Holiday.

BY A PUPIL MIDWIFE.

If a midwifery pupil is anxious to gain plenty of experience, crowded into a short space of time, I strongly advise her to arrange the period of her training in a London lying-in hospital, to include a Bank Holiday, at which time, I think, even the most enthusiastic and energetic of nurses will find sufficient work to satisfy them. Of course, it was a well-known fact that Saturday nights and Sundays we always expected to have far more in- and out-papients than any other time in the week, and consequently I always looked forward to those days, but they did not nearly come up to a Bank Holiday. Being a senior pupil, no doubt I had fully my share of cases at the particular time I am speaking of (though there were no complaints heard of slackness of work from any one), and perhaps it may interest a few "would-be midwives" to know how I spent it.

### An Interrupted Supper.

We had been particularly busy all day Saturday, and I was just sitting down to a well-earned supper when a message came from matron to say she wished to speak to me. I racked my brain to think what offence I had committed, but could not remember any particular sin at that moment, and felt very relieved when I found she only wanted to tell me to go at once to a woman on district whom I had been attending, and had recently discharged. She was a phthisical patient in a bad state of health altogether, but her confinement had been a perfectly normal one, and she had made an excellent recovery, so I was surprised at her sending for me. However, I went off at once, and was able to very much relieve her, though more by talking and sympathy than actually doing much, as it was hardly a case for me then. I was able to leave her about 11, and returned to my delayed supper, after which I was not sorry to get to bed. It seemed to me I had hardly closed my eyes when I heard the night nurse at my bedside saying, Miss A——, you are first out to-night, and here is a letter for an out-case. I sat up very sleepily and glanced at the familiar paper held out to me, and read in the top left-hand corner, "Sixth child," which was quite sufficient to make me hurry out of bed. Whilst getting into my clothes I inquired where I was to go, and was pleased to hear it was close at hand. In five minutes I was dressed, and, thanks to the night nurse, had swallowed a cup of tea, and was out in the street with my patient's husband.

### A Patient in a Stable Loft.

We very quickly reached our destination, which I discovered to be a loft over a stable. To get to it we passed through a room in which were four men asleep. My patient's room opened out of this, and I found it occupied, not only by her, but by five children of all ages up to 12, and three women, two of whom I requested to leave, and asked the remaining one to keep the children quiet. Alas! I found the baby had just been born before I arrived, which meant a B. O. A. for me to enter on my paper afterwards, instead of being able to include it in one of my requisite 20 cases for my L. O. S. exam. already looming in the near future. There seemed, as usual, very little of anything in the room to be found to make the woman and baby comfortable, but "Necessity being the mother of invention" I was able to leave her in an hour's time as comfortable as it was possible under the circumstances, and most happy and grateful to me. Once more I was delighted to get back to my bed, and slept soundly until it was time to get up to 8 o'clock breakfast. All day Sunday it did not require any more "in-cases" to make us busy, as already every bed in the wards was full, but the cry was "Still they come," and temporary beds and private rooms had to be prepared, so that we were all kept well going until supper time once more came round.

### Another Lost Supper.

Again, for me, it was a case of "There's many a slip" etc., for at the same moment that my plate was passed to me I received another of those familiar "out-case" letters, and the two words written at the top, "Ninth child," were quite sufficient for me to leave my supper untasted, tear up to my room for my bonnet, cloak, and bag, and to be in the street in less than three minutes. The man who came for me kept assuring me it was not 10 minutes' walk, but it seemed to me we walked miles and miles. In fact, I discovered the next day it was considerably out of our radius. At the end of half an hour's very fast walking, by which time I was intensely hot and rather tired, we arrived at the house in which the patient lived. I found her room was underground, but fairly clean, though only about six feet square, and a roaring fire in the grate. Once more I was greeted with the cry, "The baby is born, Nurse. He was born just 20 minutes ago," and they seemed surprised I did not seem more pleased to hear it, not knowing what another B. O. A. meant to me. There was nothing to do but to make the best of it, so after expressing the placenta which refused to be expressed for some time, I made my patient clean and comfortable, watched the nurse I had brought with me wash and dress the baby, and then we once more took our departure, leaving another very grateful patient behind us. We walked back to the hospital at a very different pace to that at which we had come, and

A BANK HOLIDAY.—*Continued.*

found our suppers and an ever-welcome cup of tea awaiting us. Between 2 and 3 A.M. I found myself once more in bed.

A Night Call.

It seemed to me I had been asleep for about five minutes, though in reality it was nearly three hours, when I heard the night nurses' well-known voice at my bedside saying once more, "another out-case for you, and the woman is very bad." Once more I scrambled into my clothes. My hair in those days did not receive a great deal of unnecessary attention, as I never dared to take it down when going to bed. The brushing of it always had to wait until dressing time for breakfast in the morning. In seven minutes the nurse whose turn it was to go out with me, was walking with me in the opposite direction to my last case; this one, I was thankful to discover, was not quite so far away. I learnt from the man who had come to fetch me to his wife that they lived in an onion loft at the back of a greengrocer's shop up an alley. To get up to their room we had to climb the very narrowest and steepest stairs I had ever seen, and that is saying something after the slums which I had been lately visiting. When we reached the top we were greeted by an enormously fat woman (so fat that up to this day I have never been able to discover how she managed to get up those stairs) who informed us that she was the lady come in to "oblige" Mrs. Brown for a bit. "She knew as how we liked to be called in in plenty of time, so she had sent for us at 5 o'clock, although, at present, Mrs. Brown pore dear, was not very bad."

Twelve Hours' Weary Waiting.

She certainly had sent in good time; for twelve tedious hours did I sit in that room where from the middle I could have touched all four walls with my hand. Twice during the day I sent my nurse back to the hospital for her meals, and to report progress, but my patient was not in a fit state for me to leave her even to go outside for a breath of fresh air, though many a time that hot summer day I felt I should have loved to. Matron remembered the bank holiday I was keeping and sent me a packet of sandwiches, but even though I occasionally felt hungry, I could not eat anything in that room where the walls and ceiling seemed positively alive. At last our patience was rewarded, and at 5 o'clock a very fine baby girl was born. The first words the mother said were: "Well, nurse, as it's a girl, I'll name her after you; that I will, whatever your name is; I don't mind, we'll put up with it." I found afterwards that this was intended as the greatest compliment they could pay for me, so I very much fear I did not appear sufficiently grateful. Soon after six I was able to leave my patient in charge of her own fat nurse, who, she assured me, was a very kind lady to her so long as she was not expected to do too much. By the time I got back to the hospital once more I was more than ready for a bath and bed, and a meal brought up to me by a sympathetic fellow pupil, after which I turned my face to my pillow and slept the clock round. I think everyone will agree with me that I had spent a somewhat busy bank holiday, but, on the whole, from my point of view, a satisfactory one.

### A Day with a District Nurse.

BY A CORRESPONDENT.

Great is the difference between the daily routine of hospital ward work and that which confronts the district nurse of a country parish. Picture a remote village in Suffolk, with a large but scattered population, some of the cottages being situated in the most out-of-the way and, to town-dwellers' eyes, inaccessible situations. The nurse starts about 9 A.M. on her morning rounds, her bag well stocked with the appliances she will be likely to need during her visits. Many district nurses have bicycles to use for their work, and they find them of great service, but districts newly started have no funds to allow of such an item just yet.

The First Case.

The first case the nurse goes to is that of a woman suffering from acute rheumatism; she has also an abscess under her arm, which the doctor opened yesterday. The patient lives in a quaint, old-fashioned cottage, which would look most picturesque and charming in a photograph, but is woefully unsuited for anybody unlucky enough to be ill in it, and the bedroom is reached by steep dark stairs. A next-door neighbour has been in already, hot water is on the fire, and everything nicely laid out, clean sheet and nightdress airing—all prepared for the nurse's visit; for she has been a constant visitor at the cottage for several days, and the people know exactly what she will need for the patient. First come temperature-taking and question asking as to pain, sleep, feeding, etc., then follows washing and bed-making (with useful help from the kind neighbour). The poultices are next renewed on the wound, for these much abused, humble applications are still greatly in favour with country doctors, and district nurses would find it hard to do without them. An hour has slipped away, a cup of hot milk is prepared for the sick woman by the nurse (no easy task to heat milk, free from "blacks," over a smoky fire-place, with a cavernous chimney overhead), then the chart is filled up and put where the doctor's eye will fall upon it, and the nurse departs.

A Great Contrast.

The next patient's room presents a far different appearance. No preparation for her visit here, as it is the first time she has been, and there are many difficulties to encounter. A small close room, no fire to speak of, a basin or towel not to be found. It is a "bad leg" which she is asked to attend to, and nurse patiently waits while numberless stockings and pieces of dirty rag are carefully removed. In answer to the question as to what remedies have been used comes a long account something like the following: "Oh! I have spent a fortune on my poor leg, shillings and 'arf-crowns on medicines and hointments and all sorts what I have been recommended to try. I am now using the 'Grasshopper Hointment and Pills,' which my brother-in-law's aunt used for her leg, and she says it has done her a lot of good, but I can't afford it. One and elevenpence ha'penny and only last a week. I can't go on any longer, so if nurse could be so kind as to tell me what to do," etc., etc. Nurse, by this time, has borrowed a basin and warm water from a neighbour who has run in to see and hear what is passing, and also to offer a little friendly advice on the treatment of bad legs, and other diseases in general. A simple cleansing dressing is soon applied, the leg neatly bandaged, strict directions given as to keeping it at rest, and showing it to the doctor, and then nurse bids the women good-bye, to hurry off on her next charge.

A Case to be Proud Of.

A baby of two, recovering from double pneumonia. This has been a wonderful case, and nurse is proud of her little patient. Fortunately the patient's home was quite close to

### A DAY WITH A DISTRICT NURSE.—*Continued.*

her abode; she could pay many visits and see that the treatment was being properly kept up. A little way on, at a cottage door, stands an anxious-looking mother. "Would nurse come in and see what is wrong with baby; she is bad, and there was no rest all night with her." Nurse notes the heavy eyes and panting breathing of the poor little mortal. Out comes the bright thermometer case from the useful bag, and there are a few quiet minutes while nurse carefully takes baby's temperature. It is 103°. You must send for doctor, and I will come in after dinner again; in the meantime keep baby warm, and give her nothing but milk till we hear what the doctor says." Nurse then goes home to dinner. She finds a message from one of the committee ladies to call when passing that way to see an old lady who has been ailing, also a note from one of the doctors to ask nurse to be present while a plaster of Paris jacket is being put on a little boy at noon the next day, if possible.

### After Dinner.

When the next round is started, nurse first visits the baby with the high temperature. She meets the doctor there, who greets her with a friendly smile: "Another pneumonia case, nurse, this is the third fresh one to-day, quite an epidemic amongst the babies just now. Left lung very bad, put the poultices here nurse," indicating on baby's back the extent of the mischief, and then turning to the mother, "send down for medicine and powder at six o'clock, I will be in again to-morrow." Off goes the busy doctor to visit his other patients for miles round. Some linseed is luckily available, "I had it for my husband when he got 'information' last year, before they sent him to the 'convulsant' home," is the astounding explanation. It would not do to laugh, so nurse has to smile inwardly, while she makes the poultice, and applies it gently to the baby, who is as "good as gold" during the process. Wool and flannel bandages are supplied from nurse's bag, and baby is left comfortably wrapped in a blanket by the fire in her little cot, while mother gets her a nice cup of milk ready.

"Don't forget to send for baby's medicine, Mrs. Brown," are nurse's parting words as she leaves her little patient for the night, hoping rather doubtfully that all will be done as it should be during her absence.

### The Troubles of District Nursing.

Thus goes the work day by day of the district nurse. Much lies on her shoulders. She must train herself never to be surprised at what she sees, or at the absence in the cottages of what seem in a hospital ward bare, absolute necessities. For she would not be welcomed as she is if she were constantly asking for things difficult or impossible to procure. She will find, in spite of teaching, entreating, and "exhortations innumerable," the people are in many cases "of the same opinion still," although they are generally too polite to say so at the time. She finds many things she would like to alter, many mistakes made from ignorance or from following bad counsel. Only by bearing in mind the golden words, "Be ye wise as serpents, but harmless as doves," can a district nurse hope to gradually and cautiously win the cottage folk to "do as nurse does" when they have sickness or injury to deal with in their homes. The important points of ventilation, changing and washing the patient, are most difficult to deal with; the greatest tact and diplomacy are needed here, but remember if you give way in trifles you are more likely to rule in great things, and sometimes a little humouring works wonders. Remember, too, the new nurse is quite unknown to her future patients; they invest her with terror; her bag even has been looked upon as containing fearful instruments of torture! An old lady who suffered with an ulcerated leg once said to her district nurse, "Do you know why I wouldn't let you come and do my leg when you first came here? well, it was because I thought you were going to 'clarify' (chloroform) me, and all the others said you carried it in your bag." District nurses will never despair, for, as the time goes on, they will find the light breaking into all the dark places, if they take their courage in both hands and work on in spite of difficulties and discouragements. It is true that great is the labour, but more than great will be the reward.

### The Amenities of Male Nurses' Co-operations.

In the King's Bench Division last week there was heard, before Mr. Justice Darling and a special jury, an action for libel brought by Mr. Michael Charles Walshe, carrying on business as the Male Nurses' Temperance Co-operation at 10 Thayer Street, Manchester Square, and at Manchester, against the Temperance Male Nurses' Co-operation, Limited, and Mr. Martin D. Gold, the secretary. The Co-operation and the defendant Gold pleaded justification of part of the alleged libel, and said of the rest that it did not refer to the plaintiff.

### The Plaintiff's Case.

In opening the case Mr. Gill said that the plaintiff had been a male nurse for eighteen years, and in 1893 he started the Male Nurses' Temperance Co-operation. Early last year a nurse in his employ named Mackenzie took out a summons at Marylebone Police Court to obtain possession of certain testimonials which plaintiff had retained. A statement was made that he was employed at £2 a week, out of which he had to pay 7s. to the plaintiff all his life. The plaintiff was not called, and the magistrate, in giving judgment for the return of the testimonials, said that his conduct was quite unjustifiable, and would amount to slavery. Next day the case was reported in two papers, one account being headed "Nurses and Slavery: Big Commissions Made Out of Their Salaries. Are Nurses Sweated?" Mr. Walshe wrote to both papers inviting the fullest inquiry, and placing his books at their disposal. They investigated them, and published statements to the effect that the plaintiff was conducting a perfectly *bonâ-fide* business, employing skilled nurses and paying them good wages. The defendant Gold had been in the employ of the plaintiff, but had left, and had started the other co-operation. In 1901 they issued a circular beginning:—

> The Temperance Male Nurses' Co-operation, Limited, was founded and incorporated to . . . do away with the sweating system which has been, and is now, practised by the various proprietary concerns trading as temperance co-operations. The proprietors of these bogus co-operations usually carry on their business under an assumed name. They charge the nurses exorbitant commissions out of their earnings, from 7s. to 13s. per week.

That was followed by the original articles that appeared in the two papers, but not a word was said of the subsequent explanations.

### Plaintiff's Evidence.

The plaintiff was called, and explained that if a nurse were employed by him at £2 a week, the charge to the patient would be £2 7s. They were not allowed to receive money or gratuities. Mackenzie had been originally an attendant at the Holborn Workhouse at £30 a year and uniform. Witness paid him over £100 a year, and he never complained that he was not sufficiently paid.

### THE AMENITIES OF MALE NURSES' ASSOCIATIONS.—*Continued.*

Cross-examined: The business was entirely witness's own. It was worked on the co-operation system.

I put it to you that the terms on which you employ these men is for them to pay you 3s. 6d. in each guinea?—The terms are that they are to receive £2 a week.

Mr. Justice Darling: Is the suggestion false?—It is.

### Defendants' Case.

Mr. Boxall, for the defendants, said that the figures gave some startling results. If Walshe had 150 men he would be getting £3,228 a year. The defendants started an organisation which was really co-operative. The question was whether this co-operation of the plaintiff's was a real thing or only an imitation. There was not a word in the circular against the nurses that the plaintiff supplied, but he was carrying on a proprietary business upon pretence of co-operation between the men.

Sir James Crichton Browne, who was called for the defence, and whose name was originally on the prospectus and was then withdrawn, said that he gave his name as patron to express his approval of the principles of temperance and training. The superintendent of the asylum at Stirling wrote him that two attendants had been employed by the plaintiff without reference to their employer, Dr. Robertson, and that they were not teetotallers. Witness therefore told the plaintiff that he could not allow his name to appear on the plaintiff's cards.

Cross-examined: Have you any opinion as to what would be a reasonable sum to pay the nurses?—That would depend on circumstances.

Did you know that Walshe was the first to start the system of having male nurses who were temperance men?—Yes.

Did you think that Gold's nurses were all members of his (Gold's) co-operation?—Yes.

I suppose a staff of 12 or 18 nurses could hardly be described as the largest in the kingdom?—I should think not.

Did Mr. Gold tell you he was a total abstainer?—No.

Mr. Martin D. Gold, secretary of the defendant co-operation, said that after being two years with the plaintiff he left his employ because Walshe did not carry out his promise with regard to the reduction of commission. John Ross was treasurer of the defendant co-operation in the first year, and in November he married a widow who had a public-house. After that he had nothing to do with the institution.

Cross-examined: Witness said he was with the plaintiff for two years and two months at £2 a week.

William Blades said that at one time he was on Walshe's staff. Witness got £2 7s. per week and paid 7s. of that to the plaintiff, except for the last case, which lasted two years, and for which he paid Walshe 10s. 6d. a week out of £3 5s. 6d. He had a case for three weeks at a guinea and half per week, and for that case he paid the plaintiff less than 7s. At no time did the plaintiff make up his salary to £2 a week. He had no agreement with the plaintiff. When witness left he asked Walshe for his testimonials, but the latter refused to give them up and still had them.

Cross-examined: Walshe required him to take the pledge, and he accordingly did so. In the case where he got a guinea and a half per week the patient was poor. When he left Walshe he continued to attend the case he then had. Recently he had put another man there. Witness never asked for payment for board and lodging. He had more than 46 weeks' employment in every year.

W. A. Creedon said that he had been on Walshe's register. There was no agreement by Walshe to pay him regular wages or to give him board and lodging. He paid Walshe 3s. 6d. in the guinea. Everybody that he heard of did the same. Walshe led him to believe that he would participate in the profit or that the commission would be reduced.

Ernest F. King, a member of the defendant co-operation, gave corroborative evidence.

Cross-examined: Did Walshe complain that you had broken the temperance pledge?—Yes, but it was not true.

Did he complain that you had used a patient's carriage to take a barmaid to the theatre?—I went with the patient and my wife.

### The Summing-up and Verdict.

Mr. Justice Darling, in summing up, said that there was no doubt that the defendant co-operation was a real co-operative society. They said there was no co-operation in the plaintiff's business, and they justified the alleged libel. The notices published by the papers about Walshe were very complimentary, but one did not wonder at that, as they were supplied by the plaintiff himself. *Truth* pointed out, after Walshe had written to them and after they had examined his books, that he and the nurses were prospering, but that to call it co-operation was absurd and misleading. The defendants said it was "bogus" co-operation, but they did not publish this second statement made by *Truth*, and the plaintiff complained that they did not; but if they had done so, would the plaintiff not have complained of it as libel? If they thought the justification was not made out they would assess the damages. The plaintiff had not tried to prove special damage to his business, but the jury were entitled to take all the circumstances into consideration.

The jury found a verdict for the plaintiff for £10 against the Co-operation and £10 against Gold.

His Lordship gave judgment accordingly.

### Everybody's Opinion.

**[Correspondence on all subjects is invited, but we cannot in any way be responsible for the opinions expressed by our correspondents. No communication can be entertained if the name and address of the correspondent are not given as a guarantee of good faith, but not necessarily for publication. All correspondents should write on one side of the paper only.]**

### TOWNS WITHOUT HOSPITALS.

"Alice Gregory" writes from the Deanery, St. Paul's, London, E.C.: I shall be exceedingly grateful if you will give publicity to my enquiries on a subject in which I am much interested. The Hospital is so widely read that I am sure some of your readers could give me information as to any small manufacturing towns where there is a growing population, and a need for a hospital for the reception of accidents and other serious cases. If anyone working in such a place will send me full particulars and statistics, I shall be deeply obliged.

### IN OFF-DUTY HOURS.

"M. L." writes: I am sure that many sisters and nurses will make good use of the hints as to where to go for enjoyment in London, described by "A Sister" in The Hospital for July 18th. I think one good turn deserves another, and as I found that a favourite spot of mine was omitted in her catalogue, it occurred to me that perhaps the writer herself did not know of it, and would be glad to hear of another interesting resort. A short distance from the Marble Arch, on the Bayswater Road, and facing the park, is a small unpretentious-looking building called "The Chapel of the Ascension." It was erected in 1890 by the wish and at the expense of Mrs. Russell Gurney, widow of the eminent Recorder of London. Her idea had been to erect in London a small chapel, after the model of one she had seen in Florence—a chapel for rest and meditation, where no services were held, but where ample food for heart and brain was provided in the form of lovely pictures, with which the walls were covered. The wellknown artist, Mr. Frederick Shields, was asked to undertake the work; and before commencing it he, in company with the architect, visited Pietra Santo in Northern Italy and saw in the principal church there the design which had originally suggested itself to Mrs. Russell Gurney for her cherished scheme at home. There is a full and most interesting account of her plan from its commencement, with a few touching incidents in her own life in the pamphlet which you obtain at the Chapel entitled "The Chapel and its Story." When I was there last the work was very nearly completed, and I was told that the artist was then engaged upon it at his residence, and that it would be finished this year. It is free and open to all, and the pictures are so lovely and so truly refreshing that I hope all sisters and nurses who have not yet been will take my advice and go the next time they are at a loss what to do when "off duty."

### ACCOMMODATION FOR NURSES.

"A London Nurse" writes Will you accord me a small space in your columns to direct attention to the necessity for improvement in the accommodation provided for nurses in hospitals, homes, and similar institutions. During recent years considerable attention has, very properly, been devoted to improving the accommodation for patients, but, except in a very few instances, such efforts have not been extended to the housing of nurses, whose treatment in general constitutes not only an injustice to the women engaged, but must operate to the distinct disadvantage of the public. Within the last few years the ranks of the nurses have been largely recruited from a class much better qualified to tend the patient and assist the surgeon than was formerly the case, and the change has been one of considerable importance to the public. Now nurses in general are neither unduly fastidious nor predisposed to complain, but the general insufficiency of their accommodation and the almost universal want of consideration for them shown by the managers of hospitals and homes must tend to prevent women of education and intelligence continuing in, and deter others from entering, a profession in which the qualifications which such women can bring to the discharge of their duties are obviously of the greatest advantage. The absence of anything like consideration for the nurse is soon seen by those who enter hospital life in the extremely long hours seven days per week, the inadequate opportunities for rest and recreation, the often serious shortcomings of the catering, and the unsatisfactory housing arrangements, too frequently objectionable in more than one way even to those quite unaccustomed to any luxurious surroundings. These defects are too often aggravated through the absence of managerial system and ordinary prevision as well as through the constant breaches of the agreements under which the probationers have entered. Of the causes of dissatisfaction and discomfort referred to, that most productive of discontent is the inadequacy of accommodation in the matter of rooms for the nurses. In hospitals the state of the institution's finances is generally advanced as the reason for such deficiencies, but such an excuse cannot apply to the numerous successful surgical homes in London, where the nurses' accommodation is frequently even more scanty and objectionable. I served over three years in a large metropolitan hospital, experiencing some of the disadvantages mentioned, as well as others, without making complaint, and I have acquired a practical acquaintance with the procedure in West End homes. In some of the latter, from which the proprietors draw substantial incomes, the sleeping apartments assigned to the nursing staff at the present time are, to describe them mildly, unsuitable for the purpose, while the nurses (from half a dozen to a dozen in one home) are unprovided with a sitting-room or any place whatever in which to receive their friends. Considering that the long hours of duty in these establishments may deprive the nurse altogether of any opportunity to go out to her friends or relatives for weeks at a time, and that the success of these institutions depends, in no small measure, on the efforts of the nurses, the latter might reasonably expect in all cases to have some waiting or sitting place other than the stairs, and for the discussion of their private affairs with friends or relatives calling to have some other alternative to the publicity of the hall than the surreptitious privacy of the bath-room. I shall not, I hope, be misunderstood as demanding or advocating luxury for the nurses, but surely they have a right to expect freedom from avoidable discomfort and some consideration in directions in which at present they receive practically none.

### The Nurses' Bookshelf.

First Aid to the Injured and Sick. By F. J. Warwick, M.B., and A. C. Tunstall, M.D.. (Bristol: John Wright and Co.; London: Simpkin, Marshall, Hamilton, Kent & Co., Limited. 1901. 216 pages. 153 illustrations. 2s. 6d.)

Dr. Warwick and Dr. Tunstall may be congratulated on having compiled a most useful little volume. It is small and sufficiently flexible to be carried in the pocket with comfort, while the print is large enough to be easily read. In their choice of matter, the authors have shown, for the most part, a wise discretion as to what to include and what to leave out. The anatomical part of the work is most comprehensive, almost too much so for a book intended for the amateur, the description of the heart muscle and of the walls of the blood-vessels being unnecessarily complete. There are, however, one or two small inaccuracies, *e.g.*, the thoracic duct is included amongst the structures within the abdomen and omitted from the list of those in the thorax, and the spleen is said to lie under the stomach. As regards physiology, only a brief sketch is required in a book on "First Aid," and the essentials are given very clearly, the description of the action of the heart and its valves being very good. It might have been as well, however, to have mentioned that the peptones and sugar, after absorption, are carried straight to the liver, as the functions of that organ must appear to the reader to be very slight. Coming to the more practical part of the book, we note that the diagnosis and treatment of fractures and dislocations are gone into most thoroughly, and the explanations are illustrated by a very large number of pictures, all of which are excellent. The subject of wounds is also well treated. The necessity of making the hands aseptic before touching a wound is, very properly, constantly repeated, but if the instructions on page 107 were strictly carried out, the patient would run some risk of dying of arterial hæmorrhage while the attendant was purifying his hands. The chapter on coma is good, and so, for the most part, is that on convulsions, but the attendant should have been told of the importance of noting whether the convulsive movements are uni- or bi-lateral or present any other peculiarities; for observations of that kind may be of the greatest use to the physician on his visit. The toxicology is very comprehensive, but the treatment of opium poisoning by Condy's fluid is omitted. Also it would have been as well to mention the late effects of phosphorus poisoning. In the matter of dog bites, the importance, in view of the Pasteur treatment, of tracing the dog and ascertaining whether it be mad, should have been inculcated.

"First Aid" to Injured and Sick. By J. F. Sutherland, M.D., Lecturer and Examiner, St. Andrew's Ambulance Association. (London: Houlston and Sons, 7 Paternoster Buildings; Edinburgh and Glasgow: John Menzies and Co. Dec., 1901. 24 pages, 2 coloured diagrams. 3d.)

This book contains a really remarkable amount of information in a very small space, and as it can be carried in the waistcoat pocket it may be recommended as a book of reference in emergencies. It would not, however, be of much use to a beginner, as many of the directions are so short as themselves to require explanation.

### Appointments.

Barrington Hospital, Limerick.—Miss Bessie Sugden has been appointed day nurse. She was trained for three years at the Royal Halifax Infirmary and has since been charge nurse of the children's ward at the General Hospital, Tunbridge Wells, and district nurse at Chester.

Bradford Children's Hospital.—Miss Hilda Cavanagh has been appointed sister. She was trained, for three years, at the Royal Infirmary, Preston, where she has since been theatre and out-patient sister.

Children's Convalescent Home, West Kirby.—Miss Hutchison has been appointed staff nurse. She was trained at the Hospital for Sick Children, Mon Edge, Newcastle-upon-Tyne.

Eastern Fever Hospital, Homerton.—Miss Clara Booth has been appointed assistant matron. She was trained, for three years, at Brownlow Hill Infirmary, Liverpool, and has been ward sister at the Royal South Hants and Southampton Hospital for eight years, charge nurse at Park Hospital, Tottenham, for five months, and since June 1900 superintendent of night nurses in the same institution.

Isolation Hospital Ottershaw, Chertsey.—Miss Joan Thompson has been appointed night charge nurse. She was trained at the Fever Hospital, Dunfermline.

Lewisham Union Infirmary.—Miss Louisa J. Buck has been appointed sister. She was trained at the Royal Hospital, Portsmouth, and has since been sister at a London medical and surgical home.

St. John's District, Keswick.—Miss Mary Tyson has has been appointed nurse. She was trained for three years at the Royal Infirmary, Sheffield, and holds the L.O.S. certificate.

St. Mary Islington Infirmary.—Miss Louisa Bond has been appointed sister. She was trained at University College Hospital, and has since been sister at the Chest Hospital, City Road, London.

Skipton and District Hospital.—Miss May Thomson has been appointed staff nurse. She was trained at Nottingham General Hospital and has since done private nursing.

Woolwich Union Infirmary, Plumstead. — Miss Margaret Annie Howes and Miss Lilly Abson have been appointed ward sisters. Miss Howes was trained, for three years, at Camberwell Infirmary, and has since been charge nurse at Holborn Union Infirmary. Miss Abson was trained at Bradford Union Infirmary, and has since been staff nurse at Wakefield Union Infirmary.

### Presentations.

General Hospital, Tunbridge Wells.—Miss Lessey, matron of Tunbridge Wells General Hospital, was last week presented with a silver tea-kettle from the nursing staff, and a silver teapot from the servants of the hospital.

St. Leonard's Infirmary, Shoreditch.—The nurses at St. Leonard's Infirmary, Shoreditch, have presented sister Crooke, who is leaving Shoreditch for Grove Hospital, Tooting, with three silver-plated flower-pot stands and a pair of sugar-tongs as a mark of their appreciation of her unfailing kindness to them.

### To Nurses.

We invite contributions from any of our readers, and shall be glad to pay for "Notes on News from the Nursing World," or for articles describing nursing experiences, or dealing with any nursing question from an original point of view. The minimum payment for contributions is 5s., but we welcome interesting contributions of a column, or a page, in length. It may be added that notices of appointments, entertainments, presentations, and deaths are not paid for, but that we are always glad to receive them. All rejected manuscripts are returned in due course, and all payments for manuscripts used are made as early as possible after the beginning of each quarter.

### For Reading to the Sick.

"IN WEARINESS BE THOU MY REST."

In weariness be Thou my Rest,
In loneliness be Thou my Friend,
In sorrow hold me to Thy breast,
And make me love Thee to the end.

*T. R.*

Let Him write what He will upon our hearts
With His unerring pen. They are His own.
. . . . . . .
Be sure He will not cross out one sweet word
But to inscribe a sweeter,—but to grave
One that shall shine for ever to His praise,
And thus fulfil our deepest heart-desire.

*F. R. Havergal.*

To let God choose for us, and to be satisfied with His choice, here is the secret of peace. "In returning and rest shall ye be saved; in quietness and in confidence shall be your strength."

*Bishop Thorold*: "The Presence of Christ."

If Peace the fruit of the Spirit is to grow within us, we must secure the abiding presence of God, not only when we are in His house or on our knees, and in times of our better moments, but always, everywhere, and in all circumstances—in times of joy and trouble, in times of sorrow and gladness, so that our nights as well as days, our darkness as well as light, may "bless the Lord, praise Him, and magnify Him for ever." And this depends on one very simple thing. We push God from us most often because we have not really learnt the simple lesson of the Lord's Prayer, "Thy will be done." If we are doing God's Will we have God as our fellow-worker, and where God's presence is there is Peace. To walk with God, in His felt presence, is to have life's troubles broken to us as they come: one day is the preparation for the next; one sorrow for another; one difficulty overcome is the help to overcome the next. Each day is met with the undivided strength which belongs to it. And He shapes our ends wonderfully and tenderly and mercifully to meet that which He sees is slowly approaching. Until we recognise at last that God's Will was the best, that His Will was the way of Peace.—*Canon Newbolt.*

The fields are white to harvest, look and see,
Are white abundantly.
The full-orbed harvest moon shines clear,
The harvest time draws near,
Be of good cheer.
Ah, woe is me!
I have no heart for harvest time,
Grown sick with hope deferred from chime to chime.
Who knocketh at His door
He welcomes evermore:
Kneel down before
That ever open door
(The time is short) and smite
Thy breast, with all thy might.
"What shall I say?"
Nay, pray.
Though one but say "Thy will be done,"
He hath not lost his day
At set of sun.

*C. Rossetti.*

### Echoes from the Outside World.

### The Movements of Royalty.

THE progress of the King's health continues to be satisfactory, and on Wednesday both the King and Queen arrived in London. They drove from Victoria Station to Buckingham Palace amid remarkable manifestations of enthusiasm. On Monday morning, for the first time since his stay on the *Victoria and Albert*, the King walked on the promenade deck above the pavilion. On the same day the Empress Eugenie paid a visit to the King and Queen, and later the Queen and Princess Victoria went on board the Royal yacht *Osborne* and made a private visit to Netley Hospital. On Monday evening Sir F. H. Laking and Dr. Bankart, at the request of the Queen, visited the late Queen's aged retainer, Mr. Stone, who lives at the Swiss Cottage on the Osborne estate and for many years has had charge of the Royal children's gardens. Mr. Stone is seriously ill and His Majesty's physicians attended him in consultation.

### The Coronation.

EVERYWHERE along the route of the Coronation procession there are fresh signs of activity, but there is a disposition to be sparing in the use of bunting, more attention being given to illuminations, which are likely to be almost as gorgeous as if the Coronation had taken place at the originally appointed time. The provincial mayors, upon telegraphing that they wished to attend, have all been invited, provided the towns over which they preside contain more than 20,000 inhabitants. In reply to the offer to send an invitation if desired, the Mayor of Clonmel sent the following reply to the Earl Marshal's invitation: "Neither wish for, nor will accept, invitation to Coronation (signed) CONDON, Mayor and M.P., East Tipperary." One of the most interesting features of the procession will be the presence of the King's Bargemaster and twelve watermen, who will look somewhat remarkable in their long scarlet coats, with gold buttons, the fronts of the coats being embroidered with the Royal Arms, with the rose, shamrock and thistle, and E R. The black velvet caps are peaked in front. The King, it will be remembered, had intended to have used the Royal Barge to go to Eton, had he not been prevented by illness, and it is at His Majesty's special desire that the bargemen are included in the procession on August 9th. They have not figured in any recent processions, having been absent both from the pageants of George IV. and Queen Victoria.

### Lord Kitchener on the Gordon College.

AT the banquet given on Friday last week by the Grocers' Company to Mr. Chamberlain and Lord Kitchener, who were also presented with the freedom of the Company, Lord Kitchener specially thanked his hosts for their donation of £1,000 to the Gordon College at Khartoum. He said he was sure that they would be glad to know that the Gordon College was making most promising progress. Lord Kitchener hopes, on his way to India, to go out to Khartoum, and to open the buildings of the college which will then almost be completed. He went on to observe, "The financial state of the college is in a very satisfactory position. We have, after a very large expenditure on buildings and on the starting of education, still some £110,000 invested in good securities, and the revenue from that sum is quite sufficient to endow the college for all its needs in the future. We shall have to wait five or ten years probably before we can see the results of the education which is now being given to the population of the Soudan; but I have no doubt of the great beneficial future that will come to that country by the action of the college."

### The Boer Leaders.

GENERAL LUCAS MEYER and his wife received an invitation from the King to witness the Coronation in Westminster Abbey; but the General, to his great regret, was compelled by "urgent medical advice" to go to the Continent immediately to take the waters. Before his departure General Meyer expressed his gratification at the warmth of his reception in England. "We came here," he said, "as strangers, but we have met the highest in the land, and been received with more friendship and hospitality than I in any circumstances could have expected." He hopes to return here in October before going back to South Africa. Mr. Steyn, accompanied by his wife and family, arrived at Southampton on Saturday morning. He seemed ill and worn, and wore blue spectacles for the protection of his eyes. He said that his doctor had forbidden him to speak more than was absolutely necessary. He wished it to be known, however, that he felt very grateful for the courtesy which he had received on board the *Carisbrooke Castle*. Dr. Pouthma, one of the chief officers of the Dutch Ambulance Corps, who was in attendance upon Mr. Steyn, and who was assisted by another doctor and five attendants, said that his patient was suffering from nervous prostration. With regard to the decision arrived at to trans-ship Mr. Steyn to the Dutch steamer *Batávia III.*, Dr. Pouthma explained that his patient could not stand the fatigue of a journey to London, so a boat had been engaged to convey him to Holland, and he could afterwards travel in an ambulance coach to his cottage.

### Social and Political.

ON Saturday morning Sir Savile Crossley, M.P., met with a serious accident while he was riding with his son on the outskirts of Somerleyton Park, near Lowestoft. Both had passed through a gate which they were about to shut when Sir Savile's Boer pony mounted the gate, and, falling back, broke the collarbone of its rider. Sir Savile also sustained a slight concussion. He has recovered complete consciousness, but suffers considerable pain in the region of the fracture, and his right thigh is badly bruised. On Sunday the Prince of Wales telegraphed for information respecting the sufferer's injuries and progress, expressing sympathy with him in his accident. Sir Savile is making excellent progress.

Last week a deputation of ladies representing the National Union of Women's Suffrage Societies waited on Sir Edward Barton to hear his opinion on the movement for Women's Suffrage in the United Kingdom. Lady Onslow introduced the deputation, which included Lady Francis Belper, Lady Henry Somerset, Mrs. Fawcett, Mrs. Lyttleton, and Miss Edith Palliser. The Premier of Australia in reply to an address which was read by Mrs. Fawcett, stated that he was himself a convert to women's suffrage. It became his duty to observe the results of it where it had been established in South Australia and Western Australia, as well as in New Zealand, and he was not able to find in those cases that the usually-predicted evils had resulted from women's suffrage. He hoped that the satisfactory consequences of the legislation in Australia would serve to dispel some of the doubts and fears which many able and conscientious men in the United Kingdom felt respecting the movement.

### Girls on Strike.

THE match girls employed at Bryant and May's have been out on strike for a week because of the introduction of a new system of work and of pooling wages. Though it seemed probable after the first few days of the dispute that a good many of the girls would arrange to give the new system the fair trial which, at any rate, it deserves, the elder women dissuaded them, and they returned in a body to their homes. But finding no disposition on the part of their employers to relinquish the disputed point, they wisely gave in after seven days of idleness. At present the new appliances have only been placed in the "Victoria" Factory, where extensive structural alterations have been going on.

### A Book and its Story.

LECTURES AT THE LOWELL INSTITUTE.*

AT a time when thoughtful readers turn for information to the works of those modern writers who seek reverently to unite the formerly alien worlds of science and religion, the issue in book form, under the title of "Through Science to Faith," of Professor Newman Smyth's Lectures, delivered in Boston, U.S.A., to the students of the Lowell Institute, will come as an able and interesting addition to other recent works on the subject. He considers, rightly, that "the too hastily conclusive and often unverified popular articles concerning religious teachings of modern science call for a painstaking and appreciative sifting of the result of modern investigation of nature in order that we may understand their real bearing upon the highest problems of human concern." For the busy person who has neither time nor the necessary scientific training to search among the sources of strictly scientific knowledge, "Through Science to Faith" will come as a help and incentive to future inquiry. Itself "the fruitage in religious thinking of these seeds gathered from the fields of scientific investigators, the author trusts that the book may be received by them as a grateful recognition from the theological side of the value of faithful scientific work, not only to the material welfare of the world, but also for the higher moral and spiritual life of men." An absence of technical expressions makes perusal an easy matter for the general reader, but for the aid of students wishing to go to the root of and inquire critically into the origin of the questions under discussion, every help is given by the aid of diagrams and reference to scientific authorities. Although not agreeing fully with the author in his view "that evolution as a general conception of the world and of the methods of life within the last fifty years of scientific observation, has advanced fully far enough to require and to justify the construction of a new natural theology, that in its turn will lead to some reconstruction of Christian theology," there is yet so much that is agreeable to views already established, and so much that lends itself to thoughtful consideration apart from points of difference, that we find every page alive with suggestive and stimulating theories starting from a scientific basis. For instance, on the question of the underlying principle of unity in creation he writes: "The naturalist who holds his science wholly apart from his faith, and the theologian who holds his faith regardless of any science, both fail to discern the underlying unity of creation. . . . Are we to regard creation as a process still going on, like an unfinished drama, or are the worlds and all things therein to be looked upon as a collection of ready-made products of all kinds and sizes like a vast department store? If we take the answer from science and say nature is not a patchwork; . . . nature is a continuous weaving of subtle unbroken threads—if, in a word, we say evolution, then the further question immediately arises, in what does its unity consist?" Not in what Haeckel in his latest book, "The Riddle of the Universe" in its "drear denial of all divinity" defines as "the simple enigma of our soulless substance," but in the solution of the problem, the ultimate mechanical and spiritual problem, which is partly scientific, partly philosophic, partly a question of fact, and partly one of interpretation, viz., that certain grand unifying principles run up and down throughout nature, and make of nature one glorious revelation." On the fact of direction in the organic world, the author writes, "If we have reason to believe that there is any providence in the great outlying world, we may look for direction also within the least cell. The internal ordering of the cell may present providence in miniature. We may scientifically apply to the divine providence the test which Jesus applied to the conduct of his disciples, and say that if it is faithful in that which is least, it will be faithful also in that which is greatest. Nor can we separate in our reasonings the problem of providence in the least and in the greatest; through the microscope and the telescope over the broad ranges of history, and in the beatings of our hearts, it is one and the same problem of the rational direction and moral guidance of life. Hence, if we would interpret the cell aright, we must not only examine it under the microscope, we must focus also upon its mystery all our light of life; and conversely, the little cell may have its contributions, not to be despised, to make to our spiritual philosophy of life." Among the, to us, most attractive chapters is that on the significance of the beautiful. The author in discussing the questions of what is the full and sufficient interpretation of the beautiful in nature, and what does its natural evolution signify? He replies, "It is the revelation of the intelligence that thinks it, and loves it, to the mind in us which may perceive it, and delight in it. This, and nothing less, is its message and its meaning. Our sciences may trace the laws of its unfolding; our biology to a certain extent may find the method of its evolution. But beauty is a perpetual revelation of intelligence to intelligence. . . . Beauty is concentrated in mind and for mind. It is not simply that ethereal waves break upon the colour purple of the eye; there would never be human sense of the beautiful should the rays of light stop in the eye: it is in the seeing mind that they are taken up, transmuted, organised into the perception and engenderment of the beautiful. Beauty has no existence except for the soul that sees it. It belongs essentially to the unseen and the eternal, although it is manifested through the passing and the seen. . . . The beautiful is expression of divinity on the face of nature. The higher interpretation of natural beauty as having rational and spiritual significance, mistakes no lines of its evolution, and comprehends any scientific knowledge of its utilities, while it does not miss the simple, divine secret at the heart of all the beauty of the world. So through the Gate Beautiful we may enter into the temple of God." In the chapter on the prophetic value of unfinished nature, in which the felt incompleteness of human work and of human aspirations and yearnings towards perfection point to the perfect life to come, Professor Smyth puts with force ideas not in themselves new, but ever fresh in their far-reaching significance. "Nature's best is nature's sure word of the coming life. . . . We must learn our song from our life. The incompleteness which we so deeply know, the strange brokenness of so much human life and love, the utter unintelligibleness to our thought and feeling of our personal life, if it has no larger sweep, no fuller joy, no heavenly freedom, all this present partialness of our truest and worthiest achievement is one grand annunciation, ever growing clearer and fuller, of the life to come, if indeed we have ears to hear nature's one deepest truth in the voice and story of all unfinished life . . . The prophecy of immortality, therefore, so far as we may read it from the evolution of individuality, is a promise likewise of social immortality . . . Only in communion with the universal life is our individuality to be made full. We lose our self-life that we may gain it in fellowship with the Father and with the Son. The life which is life indeed, is fellowship with the human and the divine. Fellowship is life's last, greatest, and immortal word."

* "Through Science to Faith." By Newman Smyth. 1 vol. Publishers: J. Clarke and Co., London. Price 6s.

### Wants and Workers.

WORKHOUSE INFIRMARY NURSE would be very thankful to receive parcels of children's clothing to distribute amongst the poor in her parish. Address "Nurse," 70 Jesus Lane, Cambridge.

NURSE SMITH would be thankful if any lady or gentleman having a spinal chair which is not further needed, would kindly give it to a bedridden patient who is needing a little open air. Address, 36 Wise Street, Dresden, Longton.

### Notes and Queries.

**The Editor is always willing to answer in this column, without any fee, all reasonable questions, as soon as possible.**

**But the following rules must be carefully observed:—**

**Every communication must be accompanied by the name and address of the writer.**

**2. The question must always bear upon nursing, directly or indirectly.**

**If an answer is required by letter a fee of half-a-crown must be enclosed with the note containing the inquiry, and we cannot undertake to forward letters addressed to correspondents making inquiries. It is therefore requested that our readers will not enclose either a stamp or a stamped envelope.**

*Home.*

(139) Can you tell me where a cripple girl of very feeble intellect could be received? It is apparently a hopeless case.—*E. M. B.*

The Hospital and Home for Incurable Children, 2 Maida Vale, might receive this case.

Can you tell me of anyone who would take a very nice girl able to work, but who suffers from nerves and makes a funny noise in her throat? Her mother would be quite willing to give the girl's services in return for a home. She has been in King's College Hospital, and the doctors say that she is not in the least mentally afflicted nor hysterical. Would you advise me to advertise?—*Mrs. B. L.*

It is quite possible that some of our nurse-readers may like to avail themselves of the girl's services in return for the patient training and watching she evidently requires to make her useful.

Will you kindly tell me where a boy born deaf and dumb and imbecile could be received permanently, free of charge?—*Nurse Eva.*

This case seems most suitable for the workhouse, but inquiry might be made at Earlswood Asylum, Redhill, or the Royal Albert Asylum, Lancaster.

Will you kindly tell me of a home near Ventnor where a consumptive patient could live after leaving the hospital. He could pay a small sum weekly.—*Nurse F.*

St. Catherine's Home, Grove Road, Ventnor, is for patients in advanced consumption. Admission is by selection and the payment of 10s. a week.

I am anxious to know if there are homes for incurable paralytic patients where a gentleman could be received for a small sum weekly.—*M. W.*

Apply for particulars of the Hospital for Epilepsy and Paralysis, 32 Portland Terrace, Regent's Park, N.W., at the National Hospital for the Paralysed and Epileptic, Queen's Square, Bloomsbury, W.C. The former receive a few paying patients, but as new premises are being built they are not ready just at present.

Will you kindly tell me of a convalescent home where a mother could take her baby, six weeks old?—*Nurse S.*

Mothers with their infants are received at the Convalescent Cottage, The Plain, Epping.

Will you kindly tell me of a home at either Margate or Ramsgate where nurses can stay and recruit their strength at small expense? *Mater.*

Either the Home of Rest for Christian Workers and Others, 39 Gordon Road, Margate, or the Y.W.C.A., apply Miss Schudt, 119 High Street, Margate, might suit you. Each charges from 15s. weekly.

Will you kindly tell me if there is a home or society which would help a poor woman of thirty-seven? She is constantly seriously ill and finds that since her mother's death she cannot keep on the house where she has maintained herself by letting lodgings. The case is a most deserving one and well known to the clergy. She is the daughter of a cabinet maker and has no relatives.—*A. H. S.*

It is an exceedingly difficult case to help. There is no benevolent fund for cabinet makers' dependents, and she is neither aged nor incurable. Try what can be done by local charities and amongst people who know her.

Can you tell me of a home where I could place an old lady who is getting childish? Her son could pay a little towards her support. *Nurse Sleeta.*

See "Burdett's Hospitals and Charities." St. Cyprian's Home for the Aged Poor, 10 Little Park Street, Dorset Square, N.W., might receive this case.

Can you tell me where the son of a clergyman, aged 44 and hopelessly paralysed, can be received for a nominal sum? He has been discharged from the hospital as incurable.—*Ella.*

As a clergyman's son he may be eligible for help from one of the following:—The Cholmondeley Charities, 2 Bloomsbury Place, W.C.; the Clergy Pensions Institution, 11 Norfolk Street, Strand, W.C.; the Friend of the Clergy Corporation, 17 King William Street, Strand, W.C.; the Poor Clergy Relief Corporation, 38 Tavistock Place, Tavistock Square, W.C.; the Society for the Relief of Clergymen, their Widows and Orphans, 8 The Boltons South Kensington, S.W.; and Tancred's Charities, 28 Lincoln's Inn Fields, W.C. As an incurable, to the British Home and Hospital for Incurables (office), 72 Cheapside, E.C.; and the Royal Hospital for Incurables (office), 106 Queen Victoria Street, E.C.

Can you tell me of a home where a girl of eleven, hopelessly paralysed, could be taken free? If there are no free institutions, will you tell me the lowest sum weekly for which she would be received, near Bristol preferred?—*Edith.*

Write to the Invalid Children's Aid Association, 12 Buckingham Street, Strand, W.C.

Can you tell me of any home where a girl of nine can be received? She is as helpless as a baby, and cannot talk, though she can hear, and gives signs of deficiency of intellect. I have written to Darenth.—*Nurse May.*

If they will not receive her at Darenth she must, we fear, be sent to the workhouse if her parents cannot maintain her.

Will you kindly tell me of a home for a woman who has given way to intemperance? She is now convalescent from a long illness.—*District Nurse.*

Apply to the Secretary, the National British Women's Temperance Association, 47 Victoria Street, S.W.

Would it be possible for me to hear of a retired nurse, who in exchange for a permanent comfortable home with bright society, would be willing to contribute 10s. a week, and give slight assistance in a case of epilepsy (attacks occur about one a year only)?—*Without Signature.*

Write to the Secretary of the Royal British Nurses' Association, 10 Orchard Street, W., where a register of elderly members is kept.

Will you kindly tell me of an institution where a woman, 58 years of age, strong and healthy, but slightly mentally afflicted, could be received? Her sister, who is a nurse, could pay 4s. a week for her maintenance.—*Nurse L. O. S.*

Write to the National Association for Promoting the Welfare of the Feeble-minded, 53 Victoria Street, S.W.

*South Africa.*

(140) 1. Where can I apply to be sent as a nurse to the concentration camps in South Africa? 2. How could I get an appointment in a Johannesburg hospital? 3. I wish to take the L.O.S. certificate; would it be any advantage for me to take the Edinburgh maternity? Can you give me any idea of the cost of taking the L.O.S.?—*Ashton and M. H.*

1. There will soon be no concentration camps. 2. Apply to the Nursing Superintendent, P.O. Box 1050, Johannesburg Hospital. 3. You can take the L.O.S. by passing the examination and fulfiling the conditions of the London Obstetrical Society, 20 Hanover Square, W. You could be prepared in Edinburgh and then present yourself for the society's examination for which the fee would be £1. See replies under "Maternity."

I am 22, and should like to go to South Africa as nurse. Please give me an idea how to apply?—*C. S.*

Probationers are trained at the Johannesburg Hospital, P.O. Box 1050. You should apply to the Nursing Superintendent, but it is doubtful if she will accept you without a personal interview. Write also to the Emigrants' Information Office, 31 Broadway, Westminster, S.W., for information about South Africa; and be careful that you have friends to take care of you both on the journey and when you reach your destination.

Will you kindly tell me where I can get information concerning nursing in the military hospitals in South Africa?—*G.*

Through the Directors of Queen Alexandra's Military Nursing Service, 18 Victoria Street, S.W.

1. Can you tell me where to apply to join the Nurses' Co-operative Society, Johannesburg, South Africa? 2. In what number of THE HOSPITAL was the establishment announced?—*T. C. and B. N. and E. L. W.*

1. Apply to the Superintendent Nurse, the Co-operation Residential Club, Johannesburg. 2. In that of June 7th.

**Standard Nursing Manuals.**

"The Nursing Profession: How and Where to Train." 2s. net; post free 2s. 4d.

"Art of Massage." (Second Edition.) 6s.

"Elementary Physiology for Nurses." 2s.

"Elementary Anatomy and Surgery for Nurses." 2s. 6d.

"Practical Handbook of Midwifery." 6s.

"Surgical Ward Work and Nursing." Revised Edition. 3s. 6d. net; post free 3s. 10d.

"Mental Nursing." 1s.

"Art of Feeding the Invalid." 1s. 6d.

All these are published by the SCIENTIFIC PRESS, Ltd, and may be obtained through any bookseller or direct from the publisher, 28 and 29 Southampton Street, London, W.C.

### Travel Notes.

By Our Travelling Correspondent.

### CV.—WHAT WE CAN SEE FOR FIVE POUNDS.

### Dieppe without its Heavy Prices.

Dieppe, once so cheap, is now quite a *ville de luxe*, and far beyond the modest means of most of us, but, being near to England, it would be very convenient, if prices were not so high, especially as the second return is only £1 6s. 3d., and the company has arranged a week-end ticket from Friday to Tuesday for 19s., which enables the head of the house to run over and see his family if they are able to remain longer than is possible to him.

But alas! £5 would go a very little way in Dieppe hotels; those palatial buildings make a merit of receiving you at 10 francs per day. Take courage, however, and follow my thrifty plan, which is to make my headquarters at Arques, four miles outside Dieppe. There are two inns—the "Château d'Arques" and the "Henry IV." If you went for a week, and were willing to occupy a small room, I think you would be taken for 6 francs, which is 5s. per day. Write to both proprietors, and ask them if they will accept those terms. I have sent so many there that I fear I have helped to raise prices.

### Arques Itself.

The chief claims of Arques to distinction are its castle and churches. The castle, nothing now but a picturesque ruin, is of the eleventh century, once no doubt impregnable. The conqueror reduced it only through starvation, when its owner, Count William, had turned it into a stronghold of robbery. It was claimed by our Henry I., but again changed hands during the captivity of Cœur de Lion, who, however, with his usual determination seized upon it on recovering his freedom. In 1204 it became definitely French. The church (sixteenth century) is very good, and has a graceful pierced parapet. Arques communicates with Dieppe by train and diligence continually, so that you can take your morning dip in the town, spend the rest of the day making an excursion to Varengeville or any of the pretty little spots round, and return to Arques in the evening.

### Dieppe—its Churches and Castle.

A very considerable change has passed over the old town in the last thirty or forty years; there is not much left that is picturesque except round St. Jacques and the fish market. Something still lingers in the part called Le Pollet, though even that is now terribly clean and brushed up. Sixteen years ago, when I saw it first, it was wonderful: a mass of narrow overhanging little streets, many of them simply *impasses* with lines stretched across from which depended the family washing, in a deplorable state of unrepair, but grateful to the eye of the artist; dingy timber houses inclining to their fall at every possible angle, their cavernous windows innocent of glass, lit up with bright pots of geranium and carnation. How the French manage their window gardening so successfully fills me with wonder and envy; never have I seen carnations anywhere else in such a flourishing state. In front, the harbour (not in its present superior condition) was full of the most picturesque craft with fishing nets of all shapes and delicious shades of brown hanging in graceful confusion from the masts; the boats were in such friendly juxtaposition to the houses, that advancing towards the dock from the land end of a street, the outlet appeared blocked by a herring boat and an intrusive bowsprit protruded upon the narrow path. But most of this is gone; Le Pollet now holds up its head with the best, and has gained much in respectability and lost much in beauty.

Round the church of St. Jacques the vegetable, fruit and flower market still holds its own, with a wealth of colour and a screaming volubility of conversation not easily surpassed. Huge yellow gourds, mighty green vegetable marrows, relieved by stacks of red tomatoes, flanked by baskets full of all kinds of flowers, still make it a charming scene and one not to be enjoyed on our side of the Channel.

The old dames, rotund of form and spotless as to coifs and neckerchiefs, make a delightful contrast to the cool grey stone of the flying buttresses of St. Jacques, into which we will enter for a few moments. It is built in what is called florid Gothic style, but it is very charming.

There are several richly ornamented chapels between the buttresses, especially one on the right-hand side, if I remember rightly near the east end; it encloses a holy sepulchre. The Lady Chapel is lovely, and the Rose window is really magnificent. For its size and style I think St. Jacques is as fine as anything in Normandy.

The church of St. Remy is of later date and not nearly so interesting, though the vast pillars are worthy of note, and there is some good work in carved statues behind the High Altar.

The castle is now used as barracks, and it is difficult to obtain permission to see the interior; but I do not think that need be regretted; it is the outside that principally claims our attention. It stands imposingly on the edge of the cliffs. In 1694 it was ruthlessly and wantonly injured, and the town almost destroyed by the English in revenge for their unsuccessful attempt upon Brest.

### Excursions Round Dieppe.

Within a walk is Varengeville (5 miles), with the old manor house of Jean Ango. It is now used as a farm house, but is in good preservation. The walls are curiously wrought in flint and stone, and so is the large dove-cot, one of the distinguishing features of the Manoir d'Ango. Large medallions adorn the entrance, among others portraits of Francis I. and Diana of Poitiers. In this building the merchant prince Jean d'Ango received with almost regal pomp Francis I., Diana of Poitiers, and many other distinguished visitors; but in the close of his life misfortune dogged him; seized for debt, he died in the Castle of Dieppe somewhere about 1550, and was buried in St. Jacques.

Just off Varengeville there was a terrible shipwreck in 1885, in spite of the noisy syren, the vibration of which one may feel at a very considerable distance.

Puys is only 1¼ mile from Dieppe in the opposite direction if you go by the sands, but this is only practicable at low tide. By the Le Pollet road it is 2½, but it makes a very pleasant afternoon excursion.

Quiberville, 8½ miles from Dieppe, can be reached by omnibus, and this will help you also to Varengeville if the walk both ways is too far. There are many other places near, but my space is filled up, and so I must leave you to find them out.

### TRAVEL NOTES AND QUERIES.

Ostend to Economise (Pauper).—Pray dismiss such an idea from your mind; it is one of the dearest places on the Continent. Perhaps you are confusing it with Bruges, which is still cheap, though not so much so as it was 20 years ago. Extreme cheapness is now only to be met with in out-of-the-way places which would not be suitable for your children. Have you ever thought of Switzerland? There are great educational advantages there, and in a modest way a little more society, though entertaining is, of course, entirely out of the question in your circumstances. The Italian towns you mention would not do whilst your children are young, because there is no school that would answer, and they would grow up purely Italian and without the elements of an English education. Later on it would be greatly to their advantage to spend a few years in Italy, and masters for language, music, and art are cheap and good. I think I should rather recommend Florence or Bologna for that purpose. Florence for preference, only there are too many English.

## Figures and Tables

**Figure f1:**
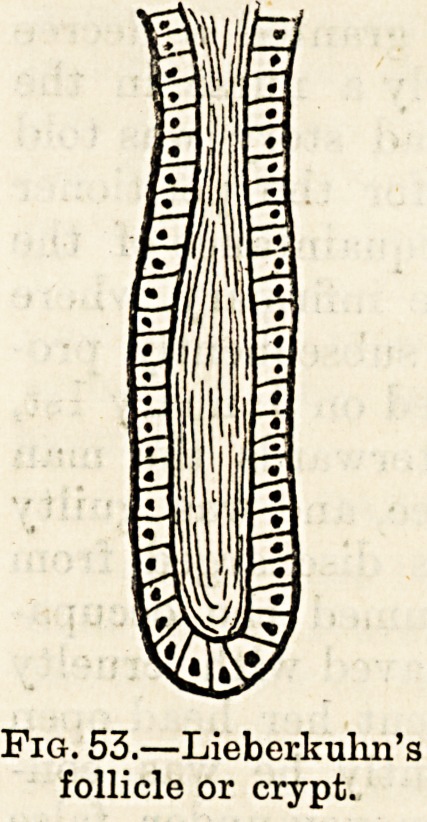


**Figure f2:**